# Supplementary material for: Holistic Motor Control of Zebra Finch Song Syllable Sequences
Source: bioRxiv. 2025 May 5:2025.05.04.652139. Preprint. [Version 1] doi: 10.1101/2025.05.04.652139 (PMC12247668; doi:10.1101/2025.05.04.652139)
Supplement: Supplement 1 [file NIHPP2025.05.04.652139v1-supplement-1.pdf]

bioRxiv preprint doi: https://doi.org/10.1101/2025.05.04.652139; this version posted May 5, 2025. The copyright holder for this preprint (which was not certified by peer review) is the author/funder, who has granted bioRxiv a license to display the preprint in perpetuity. It is made available under aCC-BY 4.0 International license.

**Supplementary video 1:**
3D rendering of zebra finch brain with tracers injected in HVC (green) and RA (red) to label efferent axons and retrogradely identified afferent neurons. RA axons flow caudally in the posterior commissure around and below Uva.

**Supplementary video 2:**
Animation displaying the model's prediction of $HVC_{PN}$ and interneuron activity waves in normal conditions, upon optogenetic stimulation, and in network degradation conditions mimicking TeNT expression in a subset of $HVC_X$ neurons.

**Supplementary video 3:**
Animation of the schematic in Fig. 6B representing a proposed simplified description of HVC dynamics in normal conditions, upon optogenetic stimulation, and in network degradation conditions mimicking TeNT expression in a subset of $HVC_X$ neurons.
